# Haptoglobin polymorphism and prostate cancer mortality

**DOI:** 10.1038/s41598-020-69333-z

**Published:** 2020-08-04

**Authors:** Melanie Kaiser, Eva-Maria Thurner, Harald Mangge, Markus Herrmann, Maria Donatella Semeraro, Wilfried Renner, Tanja Langsenlehner

**Affiliations:** 1 Clinical Institute of Medical and Chemical Laboratory Diagnostics, Medical University of Graz, Auenbruggerplatz 15, 8036 Graz, Austria; 2 Department of Therapeutic Radiology and Oncology, Medical University of Graz, Graz, Austria

**Keywords:** Cancer, Genetics, Molecular medicine, Oncology, Urology

## Abstract

Prostate cancer is a common malignancy in men worldwide and it is known that oxidative stress is a risk factor for cancer development. A common functional haptoglobin (Hp) polymorphism, originating from a duplication of a gene segment spanning over two exons, results in three distinct phenotypes with different anti-oxidative capacities: Hp1-1, Hp1-2, and Hp2-2. The aim of the study was to investigate the relationship between this Hp polymorphism and prostate cancer mortality. The study was performed on 690 patients with histologically confirmed prostate cancer, recruited between January 2004 and January 2007. Hp genotypes were determined by a TaqMan fluorogenic 5′-exonuclease assay. Hp1-1 was present in 76 (11%), Hp1-2 in 314 (45.5%), and Hp2-2 in 300 (43.5%) patients. During a median follow-up of 149 months, 251 (35.3%) patients died. Hp genotypes were not significantly associated with higher overall mortality (HR 1.10; 95% CI 0.91–1.33; p = 0.34). This remained similar in a multivariate analysis including age at diagnosis, androgen deprivation therapy, and risk group based on PSA level, GS, and T stage (HR 1.11; 95% CI 0.91–1.34; p = 0.30). We conclude that the common Hp polymorphism does not seem to be associated with overall mortality in prostate cancer patients.

## Introduction

Prostate cancer is the second most common malignant tumor and the fifth leading cause of cancer-related mortality in men worldwide^1^. Although the etiology of prostate cancer is still not entirely understood, heredity, age, and ethnicity have been firmly established as risk factors^2^. In addition, it is known that reactive oxygen species play a role in malignant transformation, progression and the aggressive phenotype of prostate cancer^3^. More precisely, the accumulation of such radicals in cells results in modification of biomolecules such as proteins, lipids, and DNA. Subsequently these alterations lead to functional impairment of the cell and diseases like cancer or cardiovascular disease.

Hydroxyl radicals, the biologically most active free radicals, can be generated when hemoglobin (Hb) breaks free during hemolysis, due to the oxidative nature of iron-containing heme^4^. Hb found in the cytoplasm of red blood cells is a functionally important protein that, amongst other tasks, carries oxygen from the lungs to the tissues of the body. Haptoglobin (Hp) is involved in promoting the clearance of plasma Hb to prevent iron loss, kidney damage and the oxidative potential of the iron contained in the Hb molecule. Binding of the Hp-Hb complex to the membrane protein CD 163, found on macrophages and monocytes, subsequently leads to clearance of the entire complex by receptor-mediated endocytosis^5^.

Beyond the task of capturing Hb in the plasma, Hp is a positive acute-phase protein which serves as a bacteriostatic agent, an inhibitor of prostaglandin synthesis and angiogenesis^6^. It is synthesized in the liver in response to inflammatory cytokines and glucocorticoids^7^. Hp is characterized by a molecular heterogeneity on chromosome 16q22 that gives rise to 3 functionally and structurally distinct phenotypes: Hp1-1, Hp2-2, and the heterozygous Hp2-1. The allelic differences originate from crossover duplication, resulting in an Hp1 allele with 5 exons and an Hp2 allele with 7 exons^8^.

The Hp1 allele product binds to hemoglobin with a higher affinity compared to Hp2, leading to a higher antioxidant capacity of Hp1^9^. The Hp2 allele results in higher B-cell and T-lymphocyte counts in the peripheral blood^10^, whereas the anti-inflammatory action is less pronounced compared to Hp1. People with Hp1-1 have the highest plasma concentrations, those with Hp2-2 the lowest, and the ones with Hp2-1 have concentrations lying in between^11^.

Due to the substantial differences in characteristics of the Hp1 and Hp2 proteins, several studies have investigated the impact of the Hp phenotype on cancer. Lee CC et al. showed an increased frequency of the Hp2-2 phenotype in people suffering from nasopharyngeal carcinoma^12^. Other studies reported an increased risk for the Hp1-1 phenotype for cervical intraepithelial neoplasia^13^ and cutaneous squamous cell carcinoma in kidney transplant patients^14^. Mandato VD et al. showed a better outcome for epithelial ovarian cancer for carriers of the Hp2-2 phenotype^15^.

For prostate cancer, no larger studies investigating the Hp polymorphism are available. Interestingly, Van Hemelrijck et al. found no association between serum haptoglobin levels and prostate cancer risk, whereas Arthur et al. reported an association of higher haptoglobin levels with increased risk of metastatic prostate cancer, but not with prostate cancer death or overall death^16,17^.

Aim of the present study was therefore to evaluate the potential association of the Hp polymorphism with long-term mortality in a large cohort of Caucasian prostate cancer patients.

## Results

HP genotypes were successfully determined in 690 (98.3%) patients of the PROCAGENE study. In the remaining 12 subjects, no valid genotype result was achieved after three attempts. All further analyses were based upon the subset of 690 patients with valid HP genotypes measurements.

Demographic data and genotype frequencies are shown in Table 1. HP genotypes were not associated with tumor stage, Gleason score or risk group. Median follow-up time was 149 months. During follow-up, 251 (35.3%) patients died.

**Table 1:** Demographic data of study participants stratified by haptoglobin (HP) phenotypes.

	Hp1-1	Hp1-2	Hp2-2	p
n	76	314	300	
Age at diagnosis (years)	67.7 ± 6.8	68.6 ± 7.0	67.8 ± 7.2	0.31
**Stage**				
T1/T2	36 (50.7)	161 (56.7)	151 (54.9)	0.66
T3/T4	35 (49.3)	123 (43.3)	124 (45.1)	
**Gleason score**				
< 7	47 (61.8)	192 (61.3)	175 (58.3)	0.71
≥ 7	29 (38.2)	121 (38.7)	125 (41.7)	
**PSA at diagnosis**				
< 10	31 (42.5)	170 (56.5)	162 (56.6)	0.16
10–20	22 (30.1)	75 (24.9)	62 (21.7)	
> 20	20 (27.4)	56 (18.6)	62 (21.7)	
**Risk group**				
Low	10 (13.2)	61 (19.4)	64 (21.3)	0.43
Intermediate	21 (27.6)	87 (27.7)	70 (23.6)	
High	45 (59.2)	166 (52.9)	166 (53.3)	
Death during follow-up	21 (27.6)	110 (35.0)	120 (40.0)	0.108

Data are presented as mean ± standard deviation, or number of subjects (percentage). Gleason score was available for 689 (99.9%) subjects, PSA at first diagnosis was available for 660 (95.7%) subjects and stage data were available for 630 (91.3%) subjects.

**Figure 1:**
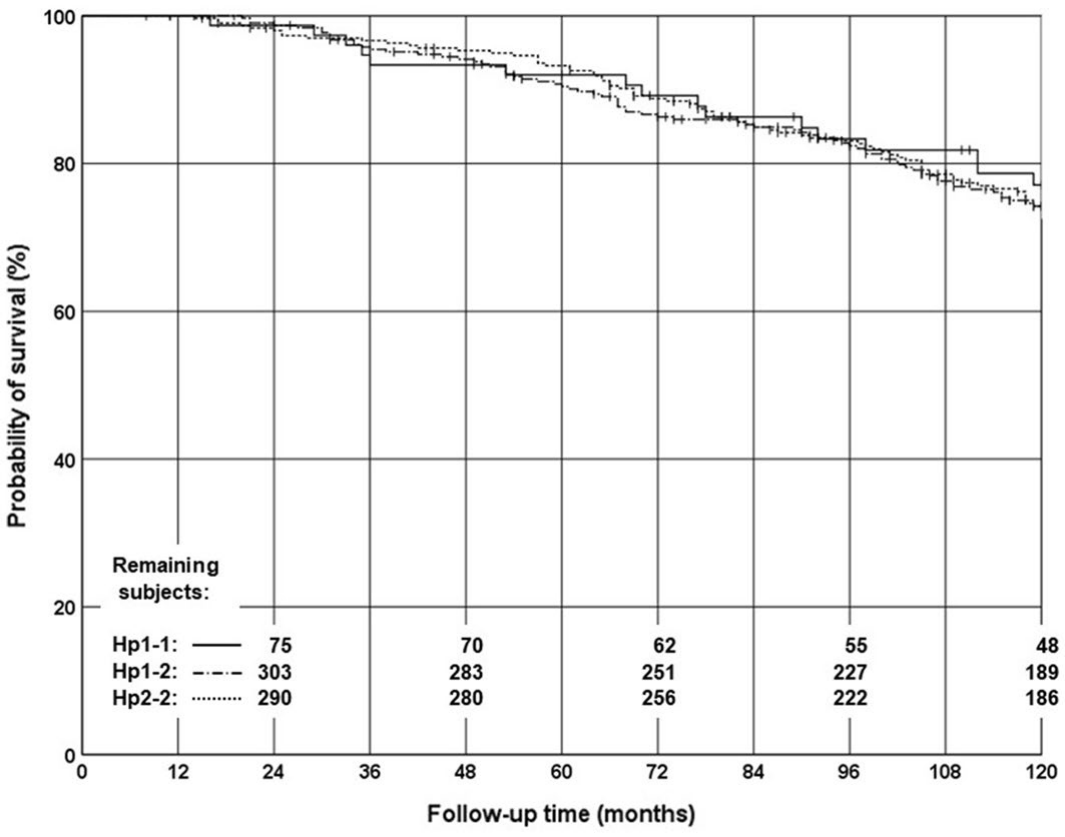
Kaplan–Meier curves of overall survival. The solid line indicates the Hp1-1 genotype, the dash-dotted line the Hp1-2 genotype, and the dotted line the Hp2-2 genotype. Vertical dashes on the lines indicate censored cases. The remaining subjects at risk in each genotype group are given in intervals of 24 months.

In a univariate Cox regression analysis, HP genotypes were not significantly associated with higher overall mortality (HR 1.10; 95% CI 0.91–1.33; p = 0.34) (Fig. 1). Similarly, in a multivariate Cox regression model including age at diagnosis, androgen deprivation therapy, and risk group (based on PSA level, GS, and T stage), HP genotypes showed no association with overall mortality (HR 1.11; 95% CI 0.91–1.34; p = 0.30).

The study had a statistical Power of more than 0.80 to exclude a HR of 1.44 or higher for carriers of the Hp2-2 genotype, and to exclude a HR of 1.77 or higher for carriers of an Hp2 allele (Hp1-2 and Hp2-2 combined).

In the majority of patients, follow-up data did not allow to discriminate between prostate cancer specific death and death from other causes.

## Discussion

On the one hand, it has been shown in previous studies that Hp polymorphism plays a role in susceptibility to certain cancers and outcome of the disease^12–14^. On the other hand, Mavondo et al. propose that Hp polymorphism is associated with neither the risk of developing prostate cancer nor outcome of disease in people of African origin. However, their study included only a brief follow up time of 18 months coupled with a small sample size. Since allele frequency of Hp1 and incidence of prostate cancer is higher in African American ancestry compared to Caucasians^18^, the study at hand explored the impact of Hp polymorphism and its prognostic value in Caucasians with prostate cancer. Therefore, Hp genotypes in 690 patients were determined with a median survival follow-up time of 149 months. The data indicate that there is no association between Hp polymorphism and overall mortality in prostate cancer patients.

The Hp polymorphism comprises a larger duplication, which is usually not captured by whole genome association studies. To the best of our knowledge there is no single nucleotide polymorphism in strong linkage disequilibrium with the Hp polymorphism, therefore no data from larger consortia are available for this polymorphism.

However, some limitations of the present study should be taken into account: No serum Hp levels were measured. Therefore, we cannot conclude that Hp levels in the blood or tissue have no effect on the survival of Caucasians with prostate cancer. In fact, Tai et al. showed that tissue Hp expression is highly correlated with better hepatocellular carcinoma tumor differentiation and increased five-year overall survival rate^19^. This still needs to be elucidated in patients with prostate cancer.

Furthermore, Goldenstein et al. suggest that the risk of developing vascular complications in Hp 2–2 individuals is likely due to the impaired ability of the Hp 2–2 protein to prevent Hb-driven oxidation. But they also found that vitamin E may be protective against cardiovascular disease in individuals comprising the Hp 2–2 phenotype^20^. However, for the present work data on the Vitamin E levels or parameter of oxidative stress were not available and thus could not be considered in the analysis.

Although our data suggest that the Hp gene polymorphism has little if any relevance for prostate cancer prognosis, this does not necessarily exclude a role of this polymorphism for other cancers. Previous studies reported associations of the Hp polymorphism with a variety of cancers, such as actinic keratosis, esophageal cancer, cutaneous squamous cell carcinoma, nasopharyngeal carcinoma, cervical neoplasia, and breast cancer^12–14,21–23^. Interestingly, for some cancers the greater risk was conferred by the Hp1 allele, whereas for others the greater risk was conferred by the Hp2 allele. The pathological mechanisms leading to these different effects of the Hp polymorphism in different cancers is currently unclear.

To conclude, this paper presents the first large scale study that provides evidence that genetic variability in the Hp gene seems not to play a prognostic role in Caucasians with prostate cancer.

## Methods

The Austrian Prostate Cancer Genetics (PROCAGENE) study included 702 prostate cancer patients who were recruited between January 2004 and January 2007. A detailed description has been published previously^24–26^. Briefly, PROCAGENE is a prospective study aimed to investigate genetic risk factors, functional relationships between genetic variations and clinical phenotypes, genetically modified response to radiotherapy (radiogenomics), and the prognostic importance of genetic markers^27–30^.

Participants of PROCAGENE were male patients with sporadic, histologically confirmed prostate cancer, treated with radiotherapy. All subjects were Caucasian. Clinical characteristics were obtained from medical records and prostate cancer patients were stratified into low-, intermediate-, and high-risk groups according to the NCCN guidelines^31^. A total of 454 patients (64.7%) received neo-adjuvant androgen deprivation therapy (ADT), 153 patients (21.8%) were treated with additional adjuvant ADT. The administration of ADT was at the discretion of the treating urologists and generally recommended in intermediate and high risk patients.

Follow-up examinations were performed in regular intervals at the Department of Therapeutic Radiology and Oncology^26^. The primary study endpoint was overall mortality.

The study was performed according to the Austrian Gene Technology Act and has been approved by the Ethical Committee of the Medical University of Graz. Written informed consent was obtained from all participating subjects. All subjects were Caucasian.

Genotypes were determined by a TaqMan fluorogenic 5′-exonuclease assay (Applied Biosystems, Austria) as described previously^32^.

Statistical analysis was done using IBM SPSS statistics 25 software (IBM Deutschland GmbH, Ehningen, Germany). Continuous variables were compared between groups by univariate analysis of variance (ANOVA). Hazard ratio (HR) and 95% confidence interval (CI) were analyzed by Cox regression analyses assuming additive effects of the HP alleles. For this analysis, genotypes were coded corresponding to the number of HP-2 alleles (HP 1/1 genotype = 0; HP 1/2 genotype = 1; HP 2/2 genotype = 2). Median follow-up times were calculated according to Schemper and Smith^33^. The criterion for statistical significance was p < 0.05.

### Research involving human participants

The study was performed in accordance with the ethical standards as laid down in the 1964 Declaration of Helsinki and its later amendments. The study has been approved by the Ethical Committee of the Medical University of Graz.

### Informed consent

Written informed consent was obtained from all participating subjects.
